# Splenic Artery Embolisation for Splenic Injury in Haemodynamically Unstable Patients

**DOI:** 10.1007/s00270-025-04138-z

**Published:** 2025-08-06

**Authors:** Patrick Brown, Naradha Lokuhetty, Panagiota Kakridas, Warren Clements, Matthew Lukies

**Affiliations:** 1 Department of Radiology, Alfred Health, 55 Commercial Road, Melbourne, VIC 3004 Australia; 2 Trauma Service, The Alfred Hospital, Melbourne, VIC 3004 Australia; 3 National Trauma Research Institute, Melbourne, VIC 3004 Australia; 4 Department of Surgery, Monash University School of Translational Medicine, Melbourne, VIC 3004 Australia; 5 Department of Medical Imaging, Monash Health, 246 Clayton Road, Clayton, VIC 3168 Australia

**Keywords:** Splenic artery embolisation, Trauma, Splenic laceration, Interventional radiology

## Abstract

**Introduction:**

Splenic artery embolisation (SAE) is a well-established treatment for high-grade splenic laceration due to blunt trauma in haemodynamically stable patients supported by major societal guidelines. However, guidelines support splenectomy in unstable patients, and there are limited data assessing the efficacy and role of SAE in this cohort. This study aimed to analyse the efficacy of splenic artery embolisation for unstable trauma patients in preventing mortality.

**Methods:**

A single-centre retrospective case–control study was performed covering a 13.5-year period. Patients with splenic laceration due to blunt trauma who underwent splenic artery embolisation or splenectomy were identified and analysed. Haemodynamically unstable patients, as defined by a shock index of ≥ 1.0 or systolic blood pressure of < 90 mmHg, who underwent SAE versus upfront splenectomy were compared as specific cohorts. The primary outcomes were all-cause 30-day mortality and splenic salvage rates.

**Results:**

A total of 126 haemodynamically unstable patients underwent SAE for blunt trauma, and eight haemodynamically unstable patients underwent upfront splenectomy. Among unstable patients who underwent SAE, splenic salvage was achieved in 98%, with 4% mortality at 30 days. Comparing unstable patients who underwent SAE versus upfront splenectomy, there was no significant difference in mortality at 30 days (p = 0.34).

**Conclusion:**

Splenic artery embolisation is a safe and efficacious treatment in unstable patients with splenic laceration due to blunt trauma, with no significant difference in mortality compared to upfront splenectomy, supporting SAE as a primary treatment standard in this patient cohort.

**Graphical Abstract:**

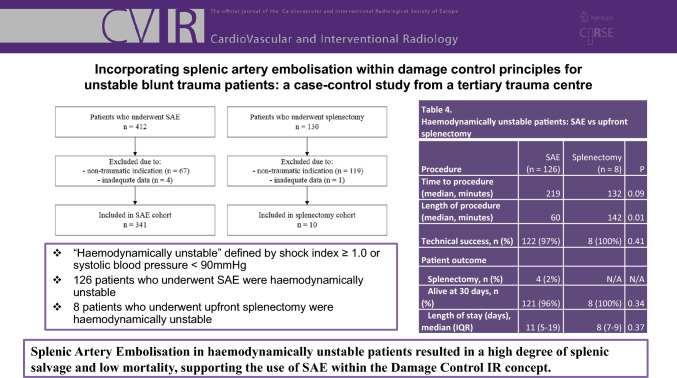

## Introduction

Splenic artery embolisation (SAE) is an established evidence-based treatment in the management of haemodynamically stable patients with acute splenic injury following blunt trauma [1–3]. By preserving the spleen, SAE mitigates the risks associated with splenectomy [4, 5]. Splenic artery embolisation has also been shown to reduce the total length of stay and associated hospital costs, when compared to surgical intervention [6–9]. The spleen plays an important role in blood cell turnover and immune function, and splenectomy is known to increase susceptibility to infections with encapsulated bacteria [10–13]. Multiple studies have investigated splenic function following SAE, with the majority confirming that the long-term immune function of the spleen is preserved [19–23].

Current guidelines recommend non-operative management in haemodynamically stable patients [4, 16, 27]. However, the application of SAE in haemodynamically unstable patients is less-studied and guidelines advocate for splenectomy, based mostly on consensus opinion [28]. Data on outcomes of SAE in unstable patients are scarce, with few studies published to date [14, 15, 27, 29].

This study aimed to analyse the outcomes of SAE in haemodynamically unstable patients following traumatic splenic injury, including all-cause 30-day mortality, splenic salvage and complications. We hypothesised that SAE is an effective treatment strategy and has a similar mortality compared with upfront splenectomy.

## Materials and Methods

### Ethics

Local ethical approval was granted (576/23), and this study is reported according to the STROBE checklist.

### Data Identification and Collection

Patients aged 16 years and over between 15 March 2010 and 6 October 2023 who underwent SAE and/or splenectomy were identified using procedure codes in the Radiology Information System (RIS) and electronic medical record (EMR). Demographics, mechanism of injury, splenic injury grade, as defined by the American Associated of Surgery of Trauma (AAST), blood transfusion volumes and injury severity scores (ISS) were collected from the EMR [30]. Procedural details, including embolic material, location of embolisation and technical success, were recorded.

### Definitions

Paired heart rate and blood pressure readings were used to calculate the highest shock index (SI) prior to intervention. Patients with an SI ≥ 1.0 or systolic blood pressure of < 90 mmHg were classified as haemodynamically unstable, which is similar to the previous studies of unstable trauma cohorts [14]. Local institutional practice is for the majority of stable or unstable patients with high grade (AAST grade 3, 4 or 5) to undergo SAE or splenectomy.

### Inclusion and Exclusion Criteria

The total pool of SAE patients (n = 341) overlaps with a previous publication (n = 232), which did not differentiate between haemodynamically stable and unstable patients [31]. Patients were excluded if they received SAE or splenectomy for a non-traumatic indication or if there were missing data about haemodynamic status, procedural success or mortality. Patients were only included in the final cohorts if haemodynamically unstable prior to intervention.

### Outcomes

The primary outcome was the all-cause 30-day mortality while secondary outcome was the rate of splenic salvage following SAE. Complications were defined according to the CIRSE classification system [32].

### Statistics

Median values were calculated for non-parametric data including time to embolisation, length of procedure and length of stay, and were compared using the rank-sum test. A probability value of less than 0.05 was considered statistically significant.

## Results

### SAE Cohort

A total of 412 patients who underwent splenic artery embolisation (SAE) were identified. After applying exclusion criteria, the final cohort was 126 haemodynamically unstable patients who underwent SAE as primary management of traumatic splenic laceration. The mean age was 42 years with a male predominance (70%) (Table 1). There was one case of penetrating trauma, with all other cases due to blunt trauma.

**Table 1:** Splenic Artery Embolisation (SAE) and Upfront Splenectomy Demographics and Outcomes

Patient characteristics	SAE (n = 126)	Upfront Splenectomy (n = 8)	*P* value
Age, mean (SD)	42 (18)	44 (20)	
*Gender, n (%)*			
Male	89 (70)	5 (62.5)	
Female	37 (30)	3 (37.5)	
*Mechanism of injury, n (%)*			
MVA	69 (55)	4 (50)	
Fall	31 (25)	2 (25)	
Other blunt trauma	25 (20)	1 (12.5)	
Penetrating injury	1 (0.8)	1 (12.5)	
Injury Severity Score (ISS, median)	28	24	
*Blood Products*			
Packed Red Blood Cells (median units)	4	4	
Fresh Frozen Plasma (median units)	3	2	
Pooled Platelets (median units)	1	1	
AAST splenic injury grade, n (%)			
1	0	0	
2	3 (2.4)	0	
3	29 (23)	0	
4	74 (59)	2 (25)	
5	20 (16)	6 (75)	
Time to operation (median, minutes)	219	132	0.09
Length of operation (median, minutes)	60	142	0.01
Technical success (n, %)	122 (97)	8 (100)	0.41
*Patient outcome*			
Splenectomy (n, %)	4 (2)	N/A	N/A
Alive at 30 days (n, %)	121 (96)	8 (100)	0.34
Length of stay (median days, IQR)	11 (5–19)	8 (7–9)	0.37

### Upfront Splenectomy Cohort

**Fig. 1:**
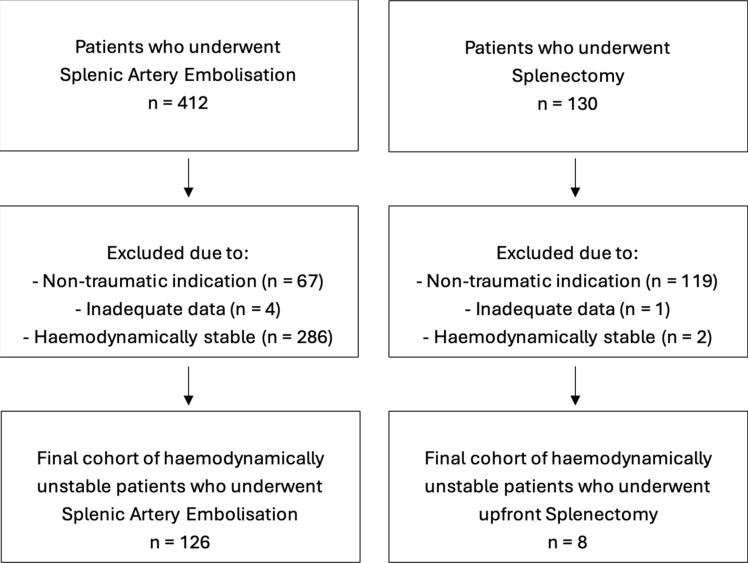
Study design flowchart

A total of 130 patients who underwent splenectomy were identified (Fig. 1). After applying exclusion criteria, the final cohort was eight haemodynamically unstable patients who underwent upfront splenectomy as primary management of traumatic splenic laceration. The mean age was 44 years, and all had high-grade splenic injuries of AAST grade 4–5 (Table 1). There was one case of penetrating trauma, with all other cases due to blunt trauma (Table 1).

### Procedural Details

The median time to intervention in the unstable SAE group was 219 min, longer than that in upfront splenectomy group (132 min) (p = 0.08) (Table 2), but largely offset by the significantly shorter procedure time for SAE (60 min) compared to splenectomy (142 min) (p = 0.01). In unstable patients who underwent SAE, contrast extravasation was seen in 58% (73/126), and petechial haemorrhage or pseudoaneurysm was seen in 80% (101/127) at the time of angiography. Most SAE procedures were performed with coils deployed proximal to the splenic hilum, with technical success achieved in 97%, defined as stasis or near-stasis of flow in the embolised splenic artery. There were no significant procedural complications with SAE, noting that the appearance of infarct on CT alone was not considered a true complication (Table 3).

**Table 2:** SAE procedure details (n = 126)

Procedure characteristic	Value
Time to embolisation (median, minutes)	218
Length of procedure (median, minutes)	60
*Type of embolisation, n (%)*	
Proximal	91 (72)
Distal	18 (14)
Proximal and distal	17 (13)
*Embolic agent, n (%)*	
Coils (only)	110 (87)
Coils + gelatin sponge	8 (6.3)
Vascular plug (only)	6 (4.7)
Gelatin sponge (only)	2 (1.6)

**Table 3:** Splenectomy post-SAE (n = 4)

Patient number	Age	Gender	AAST injury grade	Location of embolisation	Type of embolic used	Reason for splenectomy	Days to splenectomy	Mortality at 30 days
1	60	F	4	Distal	Coils	Increasing abdominal pain	1	Yes
2	48	F	5	Proximal	Coils	Re-bleed	4	No
3	32	M	5	Proximal	Coils	Re-bleed	4	No
4	37	F	4	Proximal	Plug	Persistent pseudoaneurysm	10	No

### Clinical Outcomes

The overall splenic salvage rate was 98%, and 30-day mortality was 4% (Tables 1 and 4). Four patients (2%) underwent splenectomy after SAE. Causes of mortality included severe traumatic brain injury, sepsis secondary to hospital-acquired pneumonia and ischaemic bowel unrelated to SAE. Comparing the unstable patients who underwent SAE versus upfront splenectomy, there was no significant difference in the all-cause 30-day survival rate (96 versus 100%; p = 0.34) (Table 1). Overall length of hospital stay was slightly longer for SAE than splenectomy (median of 11 versus 8 days), which may reflect the higher median ISS score in the SAE cohort (28 versus 24).

## Discussion

Despite its established role in stable patients, the application of SAE in haemodynamically unstable patients remains less studied and consensus-based guidelines advocate for splenectomy [28]. Our study demonstrated high rates of technical success and splenic salvage, with comparable 30-day survival to upfront splenectomy. The splenectomy rate of 2% in our study was lower than 29% of Guinto et al. and 12% of Zoppo et al. in unstable cohorts [14, 15]. These differences may be due to factors including smaller sample sizes and differences in resuscitation and IR practices within the multidisciplinary trauma team. The mortality rate in our unstable SAE cohort was low (2%) and consistent with Zoppo et al. [14], who demonstrated no significant difference in 30-day survival rates comparing stable and unstable cohorts. The study by Zoppo et al. did not, however, have a comparison with upfront splenectomy.

Our local institution primarily utilises embolisation as the first-line treatment for AAST grade 3–5 splenic injuries on CT, regardless of angiographic findings at time of DSA, with upfront splenectomy typically reserved for those patients who are already planned for laparotomy for other indications. Embolisation is typically performed by deploying coils proximal to the splenic hilum. Patients are monitored after SAE clinically, then discharged and followed up in the IR outpatient clinic after at least 6 weeks with testing of Howell–Jolly bodies. The IR unit is staffed by seven specialists (5.5 effective full-time) who provide 24 h on-call services.

Our study has limitations to acknowledge, including the single-centre retrospective design that may lack some items of clinical data and have limited broad applicability between centres. The measurements used to indicate haemodynamic instability, namely systolic blood pressure and shock index, are imperfect and may not account for transient responders or permissive hypotension. There was also variation in procedure technique, including embolisation agents and location.

## Conclusion

This study showed that SAE in haemodynamically unstable patients resulted in a high rate of splenic salvage and low mortality, with no significant difference in mortality compared to a small cohort of unstable patients who underwent upfront splenectomy, supporting SAE as a primary treatment standard in this patient cohort.
